# Plasticity of differentiated cells in wound repair and tumorigenesis, part I: stomach and pancreas

**DOI:** 10.1242/dmm.033373

**Published:** 2018-07-23

**Authors:** Joseph Burclaff, Jason C. Mills

**Affiliations:** Division of Gastroenterology, Departments of Medicine, Pathology and Immunology, and Developmental Biology, Washington University, St Louis, MO 63110, USA

**Keywords:** Dedifferentiation, Plasticity, Regeneration, Stem cells, Tumorigenesis

## Abstract

For the last century or so, the mature, differentiated cells throughout the body have been regarded as largely inert with respect to their regenerative potential, yet recent research shows that they can become progenitor-like and re-enter the cell cycle. Indeed, we recently proposed that mature cells can become regenerative via a conserved set of molecular mechanisms (‘paligenosis’), suggesting that a program for regeneration exists alongside programs for death (apoptosis) and division (mitosis). In two Reviews describing how emerging concepts of cellular plasticity are changing how the field views regeneration and tumorigenesis, we present the commonalities in the molecular and cellular features of plasticity at homeostasis and in response to injury in multiple organs. Here, in part 1, we discuss these advances in the stomach and pancreas. Understanding the extent of cell plasticity and uncovering its underlying mechanisms may help us refine important theories about the origin and progression of cancer, such as the cancer stem cell model, as well as the multi-hit model of tumorigenesis. Ultimately, we hope that the new concepts and perspectives on inherent cellular programs for regeneration and plasticity may open novel avenues for treating or preventing cancers.

## Abstract (precis)

**Summary:** Epithelial cells in diverse tissues have an innate plasticity program (recently dubbed ‘paligenosis’) such that differentiated cells can become regenerative upon large-scale insults to an organ. Thus, the proliferating, regenerative cells in an organ may not always derive directly from stem cells, which invites us to refine theories about the cell of origin of cancer such as the multi-hit model of tumorigenesis.

## Introduction

The series of sequential cell fate choices governing how normal, adult differentiated cells arise from their precursors has been well delineated over the last decades. The opposite process, in which cells dedifferentiate to reacquire progenitor properties, though noted by pathologists over a century ago (Adami, 1900) and demonstrated by occasional, pioneering studies (Box 1), only reentered the scientific mainstream a decade ago, when Yamanaka and others demonstrated that multiple adult cell types can be induced to return to pluripotency (see Box 2 for a glossary of terms) (Takahashi and Yamanaka, 2006). Since then, research has expanded to also examine the native capacity of mature cells *in vivo* to reverse their differentiated state in nearly all tissues (Mills and Sansom, 2015; Tata and Rajagopal, 2016). The plasticity of cells in a tissue manifests in multiple ways: stem cells (SCs) can interconvert to other SC populations, mature cells can dedifferentiate to recapitulate the earlier stages of their ontogeny, and mature cells can transdifferentiate to mature cell types of different lineages (Jopling et al., 2011).

**Box 1. Cell plasticity: a historic perspective**

Biologists observed cellular plasticity in various animal models long before the advent of genetic approaches (Brockes and Kumar, 2002; Singh et al., 2010). The earliest studies began with observations of natural regenerative abilities in animals, with Thevenot, Du Verney and Perrault demonstrating lizard tail regeneration in 1686 (described in manuscript form in Thevenot et al., 1733) and Spallanzani – who also did pioneering stomach studies (reviewed in Saenz and Mills, 2018) – reporting salamander limb regeneration in 1768 (Spallanzani, 1768). This was followed by experiments showing that amphibians of the order Urodela, including newts and salamanders, can regenerate retinas and lenses (Wachs, 1920; Stone and Chace, 1941) as well as jaws and the olfactory apparatus (Vallette, 1929). Studies became increasingly focused on the mechanisms driving this regeneration, with the idea that the mesoderm dedifferentiates to mediate the repair appearing by the mid 1900s (Chalkley, 1954).

The mid-twentieth century saw the advent of plasticity research at the cellular level, starting with nuclear transfer experiments in frog eggs. Studies through the 1950s had shown that the nucleus from a blastula cell could be successfully transplanted into an enucleated egg and grown to a tadpole (Briggs and King, 1952) and that nuclei from other early developmental states were also viable (Gurdon, 1960). In 1962, John Gurdon demonstrated that nuclei from a fully differentiated intestinal cell from feeding tadpoles was competent to form a full tadpole when transplanted into an enucleated egg (Gurdon, 1962).

Experiments on natural regeneration eventually expanded to include many organs and species, including the zebrafish heart (Poss et al., 2002) and the skin, kidney and Schwann cells of mice (Cai et al., 2007). Studies have also become increasingly mechanistic, culminating in the discovery of distinct factors necessary and sufficient for the reprogramming of differentiated cells to a pluripotent state (Takahashi and Yamanaka, 2006).

**Box 2. Glossary**

**Astrocytes:** glial cells of the central nervous system, characteristically with a star-like morphology.

**Cerulein:** a hyperactive analog of the pancreatic secretion-inducing hormone cholecystokinin (CCK), causes pancreatic injury upon injection.

**Dysplasia:** the presence of abnormal cell types in a tissue that carry clear risk for progression to cancer.

**Endocrine:** cells that secrete hormones into the circulation.

**Exocrine:** cells that secrete proteins away from the body (e.g. into the lumen of the gastrointestinal tract).

**Gastritis:** inflammation of the stomach lining.

**Granules:** small compact particles of substances within (secretory) vesicles in cells.

**Haploinsufficiency:** when a phenotype manifests due to loss of one wild-type allele of a gene.

***Helicobacter pylori*:** gram-negative bacterium that colonizes the stomachs of over 50% of the world's population (Amieva and Peek, 2016). In some people, *H. pylori* cause inflammation with loss of parietal cells and metaplastic alteration of chief cells, eventually leading to gastric cancer.

**Intestinal metaplasia:** a pattern of reaction to injury wherein the differentiation pattern of small or large intestinal epithelium develops within other organs.

**Lineage tracing:** experiments to determine all progeny from a specific cell by using cell-specific promotor genes to express reporter genes only within target cells and their progeny.

**Lumen:** the space that is lined by an epithelium (e.g. the cavity of the stomach where food begins to be digested).

**Metaplasia/metaplastic cells:** the process wherein otherwise normal cells appear in the wrong tissue setting.

**Nucleotide tracing:** administering nucleotides tagged with a trackable marker to monitor cells which were actively synthesizing DNA at the time of administration.

**Pancreatitis:** inflammation of the pancreas.

**Pluripotency:** term for an undifferentiated cell with the potential to become any cell in the body.

**Quiescence:** when a cell is not actively cycling (e.g. remains in the G0 stage of the cell cycle).

**Ras genes:** gene superfamily encoding for small GTPase proteins which transmit signals when activated, often promoting genes involved in cell growth and survival. *HRas*, *KRas* and *NRas* are commonly mutated in human cancers (Downward, 2003).

**Schwann cells:** cells of the peripheral nervous system that produce myelin sheaths around neuronal axons.

**Stochastically:** randomly determined.

**Zymogenic:** term for a cell producing zymogens, inactive substances that are converted to digestive enzymes.

Cellular plasticity may be key to regeneration upon large-scale injury, yet a tissue's capacity for plasticity may also carry an inherent potential for adverse consequences, such as cancer. Here, we discuss how plasticity may help refine a long-standing model for how cancer begins. The well-established ‘multi-hit model’ postulates that tumors arise when long-lived SCs accrue mutations necessary for tumorigenesis (Fearon and Vogelstein, 1990). Recently, though, it has become clear that individual SCs in mice may not be as long-lived as traditionally believed (Lopez-Garcia et al., 2010; Snippert et al., 2010; Baker et al., 2014), raising the question of how a single SC could accumulate multiple mutations over the course of years (Mills and Sansom, 2015). Even if the SC population remains stable over time, intestinal SCs are relatively short-lived, as SCs divide frequently and stochastically (Box 2), commonly jostling each other out of the niche in mice (Lopez-Garcia et al., 2010; Snippert et al., 2010) and in humans (Baker et al., 2014). Although some intestinal SCs tend to be longer-lived (Ritsma et al., 2014) and SCs with oncogenic mutations hold a competitive advantage over wild-type SCs in the intestinal crypt (Snippert et al., 2014), the question remains whether SCs are the sole population that accumulates tumor-inducing mutations over the lifetime of an organism. Moreover, in organs such as the pancreas that lack a constitutive SC, other cell types must accumulate such tumor-inducing mutations.

Increasing evidence shows that plasticity can be involved in the origin of cancers in numerous epithelial tissues (Giroux and Rustgi, 2017) and even astrocytes (Box 2) (Friedmann-Morvinski and Verma, 2014). This Review highlights the diversity of cell types that may accrue the ‘multiple hits’ defined by Kinzler and Vogelstein (Vogelstein and Kinzler, 1993) and initiate tumor formation. A more complete understanding of the process of mutation accumulation may further improve our understanding of how every organ produces tumors with a multitude of phenotypes that vary not only from person to person but even within a single person: tumors initiated by SCs or by cells at various stages of differentiation or dedifferentiation may contribute to this diversity (Visvader, 2011; Song and Balmain, 2015).

**Fig. 1.**
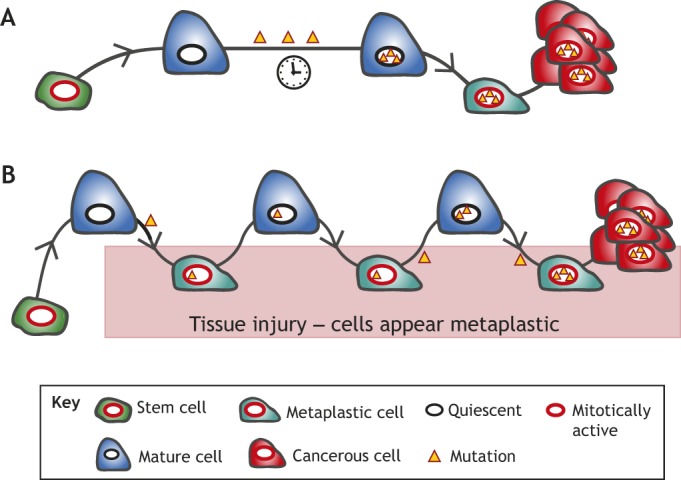
**Proposed models of mature cells acting as cancer cells of origin.** We propose that long-lived mature cells may accumulate and store mutations, eventually acting as – or giving rise to cells that can act as – cells of origin for cancers in diverse tissues. This mutational accumulation may occur in two main ways: (A) mature cells (dark blue) may accumulate mutations (yellow triangles) as they maintain their mature functioning cell fate over time. The mutations themselves or stressors may trigger dedifferentiation (teal cell). If the acquired mutations are sufficiently carcinogenic, they may then block the cell in the dedifferentiated state, causing it to expand as a clone that can give rise to cancer (red). (B) The ‘cyclical hit’ model describes mature cells that dedifferentiate and redifferentiate multiple times in response to injury/inflammation. Each time the cells are called back into the cell cycle, replicative stress can promote mutation accumulation. Differentiated cells can store such mutations indefinitely. Eventually, a mutation or combination of mutations is sufficient to block the cell in one of its replicative phases and lead to clonal expansion and potential tumorigenesis.

Plasticity can allow post-mitotic cells to re-enter the cell cycle, and we have proposed that cycles of proliferation and quiescence (Box 2) can favor tumorigenesis because accumulated mutations can become fixed in long-lived differentiated cell populations. We have termed this the ‘cyclical hit’ model, in which cell lineages cycle through phases of dedifferentiation and redifferentiation, allowing for the accumulation and unmasking of mutations in long-lived cells (Fig. 1) (Mills and Sansom, 2015; Saenz and Mills, 2018).

In part I of this Review, we survey the current state of plasticity research in the stomach and pancreas, both of which experience the recruitment of long-lived, mature secretory cells back into the cell cycle upon certain types of physiological injury. We discuss how recent advances in our knowledge of these events and their governing mechanisms address how mature cells might initiate or be involved in tumorigenesis, challenging the idea that adult SCs are the sole cell type responsible for both accumulating mutations and spawning cancers (White and Lowry, 2015). We end part I by exploring the similarities between the responses in the two organs and postulate that they might be governed by conserved cellular programs, which hold important implications for cancer initiation. Our analysis will be continued in part II of this Review (Burclaff and Mills, 2018), where we discuss recent studies highlighting plasticity in the skin and the intestine, and explore overall similarities in plasticity and tumorigenesis across all four organs. Although the cell fate changes and reprogramming that occur during epithelial-to-mesenchymal transition are also important examples of cell fate changes with cancer implications (Varga and Greten, 2017), they are outside the scope of our Review, which will be confined to cell-autonomous processes within cells that begin as and remain epithelial cells, even as they become neoplastic.

## Stomach

**Fig. 2.**
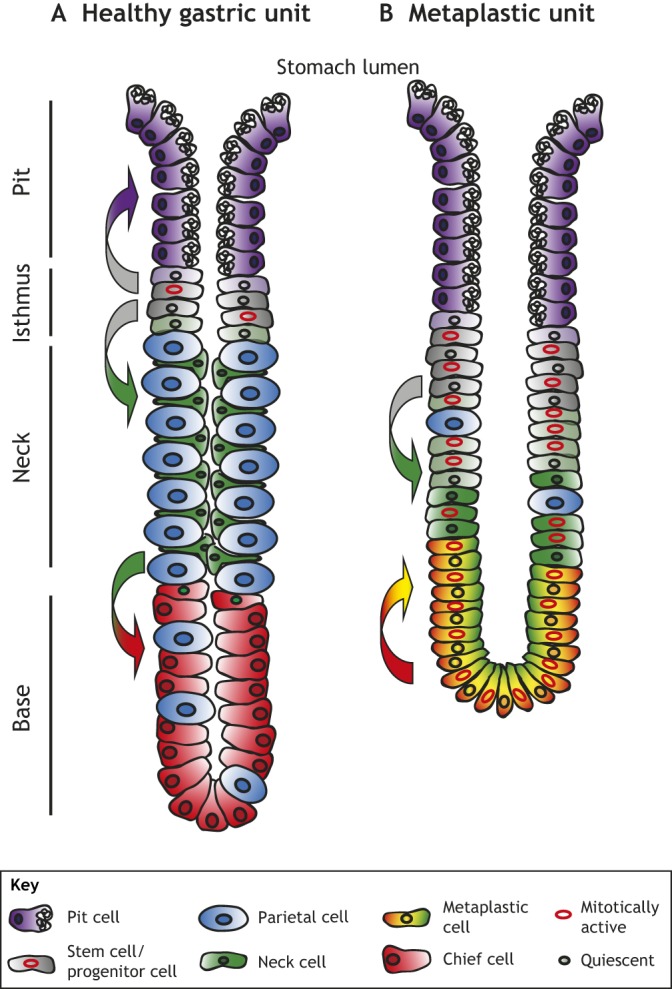
**The gastric unit and its response to injury.** (A) The healthy gastric unit, with pit cells at the opening to the gastric (stomach) lumen (Box 2), stem cells at the isthmus, parietal cells and neck cells in the middle of the unit, and chief cells at the base. Not pictured: endocrine and tuft cells. Proliferation (red nuclei) is confined to the isthmus, with new pit cells migrating upwards and parietal and mucous neck cells migrating downwards. Neck cells transition to chief cells at the zone between the neck and the base of the gastric unit. Colored arrows mark the direction of cell changes. (B) A metaplastic gastric unit after injury, such as by *Helicobacter pylori* infection or acute pharmacological agents. Parietal cells quickly die and mature chief cells become metaplastic cells co-expressing chief and neck cell markers. Proliferation occurs from the isthmus through the base, with paligenotic (capable of dedifferentiation) chief cells re-entering the cell cycle.

The stomach body (corpus) is lined by an epithelium that is flat on the luminal (see ‘Lumen’, Box 2) surface but invaginates into glands descending towards the musculature. The gland and its surface epithelial cells form the gastric unit, which contains mucus-secreting surface pit foveolar cells at the surface, mucous neck cells interspersed between acid-secreting parietal cells in the neck region (Bredemeyer et al., 2009), and zymogenic (Box 2) chief cells at the base (Karam and Leblond, 1993a,b,c,d) (Fig. 2A). Proliferation in the healthy gastric epithelium is overwhelmingly confined to morphologically undifferentiated cells located above the neck cells at the isthmus of the unit. Based on their ultrastructure and on nucleotide tracing (Box 2) studies (Hattori and Fujita, 1976; Mills and Shivdasani, 2011), these isthmal cells have long been assumed to be multipotent SCs that fuel the replacement of all mature cells in the gastric unit, although the extant data do not rule out other self-renewal mechanisms (Bjerknes and Cheng, 2002; Quante et al., 2010; Willet and Mills, 2016; Wright, 2016).

Although several candidate SC markers have been identified in cells that populate the gastric unit (Phesse and Sansom, 2017) (Table 1), none of these have been shown to be enriched exclusively in the isthmal cells. This means that a verified marker of gastric epithelial SCs in the body of the stomach remains to be identified.

**Table 1.**
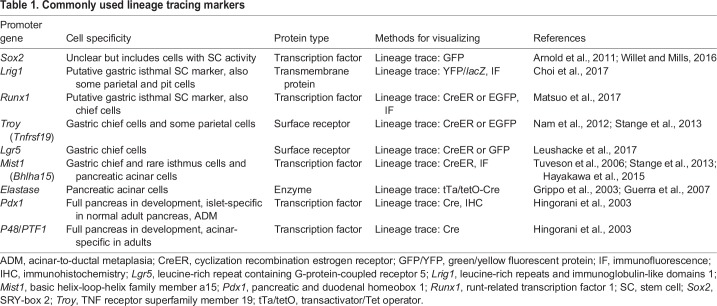
**Commonly used lineage tracing markers**

### Gastric plasticity

Chief cells are large, long-lived and non-proliferating cells that devote their energies to producing digestive enzymes. However, surprisingly, chief cells in both mice and humans are plastic: they can disassemble their complex secretory apparatus (Capoccia et al., 2013; Lo et al., 2017) to re-enter the cell cycle and, in some cases, potentially act as reserve SCs upon injury (Stange et al., 2013). In humans, *Helicobacter pylori* (Box 2) infection can cause chronic atrophic gastritis (Box 2). In this condition, parietal cells die (atrophy) and increased proliferation is observed among the remaining cells in the gastric unit. In mouse models, both *H. pylori* and various drugs can be used to kill the parietal cells and force the recruitment of other cells as additional reserve SCs (Sigal et al., 2015; Petersen et al., 2017b). Drugs that mimic the *H. pylori*-induced cellular changes include high doses of the selective estrogen receptor modulator tamoxifen (Huh et al., 2012; Saenz et al., 2016), the neutrophil elastase inhibitor DMP-777 (Goldenring et al., 2000; Nomura et al., 2005) and its ortholog L635 (Weis et al., 2013). In all cases, the observed changes include loss of parietal cells, loss of mature chief cells and the emergence of metaplastic cells (Box 2). In the stomach, the metaplastic cells that emerge upon parietal cell death express large amounts of trefoil factor 2 (TFF2; also known as spasmolytic polypeptide), so the cell lineage shifts in chronic atrophic gastritis have been called spasmolytic polypeptide expressing metaplasia (SPEM).

SPEM cells were originally thought to arise via proliferation from the isthmal SCs undergoing an alternative differentiation path, and some continue to believe that to be the case (Hayakawa et al., 2015, 2017; Kinoshita et al., 2018), yet lineage tracing (Box 2) studies with multiple genetic drivers (Table 1) from the base and isthmus of the gastric unit in mice, with corroboration in human tissues, indicate that the majority of SPEM cells, at least in the acute setting, likely arise from chief cells that reprogram to express TFF2 and re-enter the cell cycle (Lennerz et al., 2010; Nam et al., 2010; Goldenring et al., 2011; Capoccia et al., 2013; Leushacke et al., 2017; Matsuo et al., 2017; Mills and Goldenring, 2017) (Fig. 2B). Gene promoters that have been used to lineage-trace chief cell reprogramming into progenitor cells include: tumor necrosis factor receptor superfamily member 19 (*Tnfrsf19*; known as *Troy*), which is mostly expressed in mature chief cells; basic helix-loop-helix family member A15 (*Bhlha15*; known as *Mist1*), which is almost exclusively expressed in chief cells; and leucine-rich repeat containing G protein-coupled receptor 5 (*Lgr5*), which are likewise almost exclusively expressed in chief cells (Table 1). Recent work further supports the interpretation that mature chief cells are the predominant source of acute SPEM cells, showing that SPEM can arise even when any potential proliferative contribution from the SC or progenitor cells is abrogated (Radyk et al., 2018). Interestingly, SPEM cells recapitulate many aspects of immature cells in the early developing stomach, where there are abundant proliferating cells that co-express TFF2 and markers of chief cell differentiation (Keeley and Samuelson, 2010). The SPEM cells are not characteristic of the adult isthmal SCs, which lack granules (Box 2) or other ultrastructural characteristics of any specific differentiated cell lineage (Box 2) (Karam and Leblond, 1993a).

Although parietal cell loss is nearly always correlated with SPEM, a recent study demonstrated that highly targeted parietal cell apoptosis alone is insufficient to induce metaplasia (Burclaff et al., 2017). The cause and mechanism of SPEM initiation remain enigmatic, although several players have been implicated, such as requirement for a signaling cascade including extracellular signal-regulated kinase (ERK), cluster of differentiation 44 (CD44), and signal transducer and activator of transcription 3 (STAT3) (Khurana et al., 2013), macrophages, and interactions between interleukins IL-33 and IL-13 (Petersen et al., 2014, 2017a). Our group's recent work also characterizes a sequential, stepwise process that chief cells use to reprogram: (1) autodegradation, (2) induction of metaplastic gene expression, e.g. SRY-box 9 (SOX9) and TFF2, and (3) cell cycle re-entry (Willet et al., 2018). Each step has checkpoints that cells must traverse to complete proper tissue regeneration. For example, blocking lysosomal functioning stopped cells from inducing SOX9/TFF2, and inhibiting mTORC1 stopped cell cycle re-entry. The stages and checkpoints were preserved in pancreatic regeneration, and additional experiments, as well as other literature, indicated that kidney and liver regeneration follow the same sequence. Thus, there is support for a conserved cellular regenerative/dedifferentiation program that has been called ‘paligenosis’, suggesting that cells, in addition to programs for cell death (apoptosis), also have programs to regain regenerative ability (Messal et al., 2018).

### Gastric tumorigenesis

Since Pelayo Correa's early work mapping the histological stages of gastric cancer progression (Correa, 1988), it has been known that patients with metaplasia/chronic atrophic gastritis have an increased risk for gastric cancer (Hattori, 1986; Kakinoki et al., 2009; Goldenring et al., 2010) and that gastric cancer seems to arise in a stepwise fashion. The stages of gastric tumorigenesis cannot be fully studied in mice, as no mouse models of gastric cancer faithfully replicate late-stage human disease (Petersen et al., 2017b). Humans with extensive metaplasia and SPEM nearly invariably also get intestinal metaplasia (Box 2), but intestinal metaplasia does not seem to be a common feature of injury response in mice. In some mouse models, however, SPEM can progress to proliferative lesions with histological abnormalities resembling human dysplasia (Box 2) (Nomura et al., 2004; Petersen et al., 2017b). The architecture of the gastric unit is beneficial for studies characterizing the initial steps of gastric tumorigenesis, as the spatial separation between the normal isthmal and injury-induced basal proliferation zones allows for inferences to be made about the cells of origin for metaplasia and dysplasia (Radyk and Mills, 2017). Multiple recent studies have shown how proliferative dysplasia can be induced solely by expressing activated Kirsten rat sarcoma (KRAS; see ‘Ras genes’, Box 2) using multiple promoters found in chief cells (Choi et al., 2016; Leushacke et al., 2017; Matsuo et al., 2017). Such studies show that chief cells can act as cells of origin for tumorigenesis. As there is no known promoter with reliable specificity for the isthmal SC in the stomach, similar direct evidence does not exist for the SC acting as another potential gastric cancer cell of origin, although this is certainly a possibility that awaits better genetic tools for study in future work. The ability of the chief cells to act as cells of origin for gastric cancer is consistent with the ‘cyclical hit’ model of tumorigenesis, whereby long-lived chief cells may accumulate and store mutations in rounds of dedifferentiation and redifferentiation in chronic inflammation or metaplasia, possibly leading to tumorigenesis (Fig. 1) (Mills and Sansom, 2015; Saenz and Mills, 2018).

## Pancreas

**Fig. 3.**
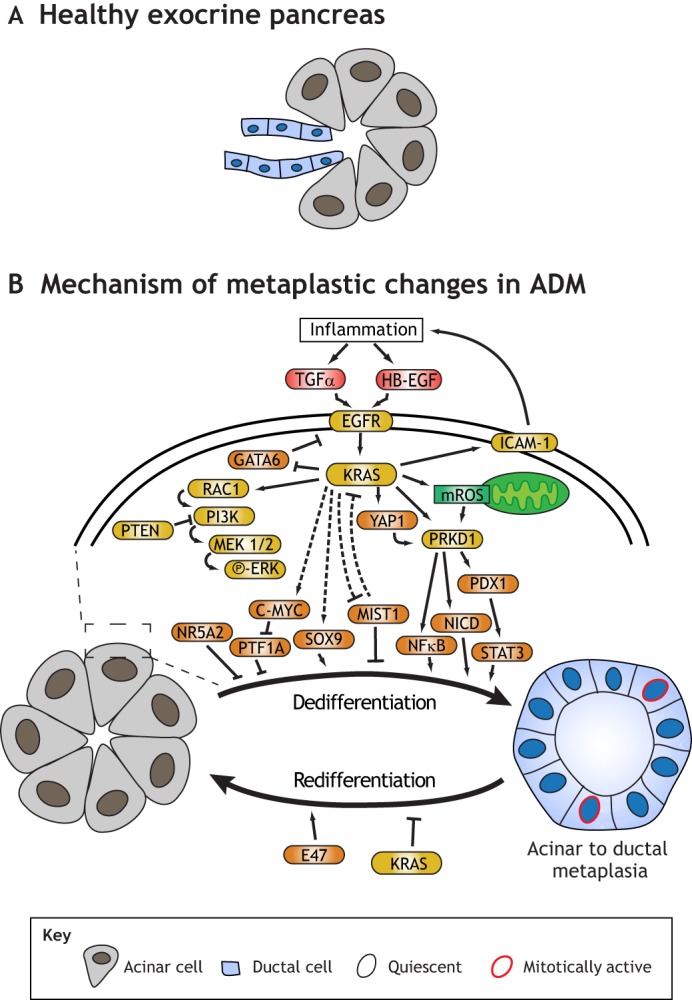
**The exocrine pancreas and the mechanistic steps involved in acinar-to-ductal metaplasia.** (A) The healthy exocrine pancreas, with acinar cells arrayed at the end of tubes lined by ductal cells. (B) Pancreatic acinar cells are normally mature and post-mitotic. Following injury with physical damage, chemical agents or expression of activated Kirsten rat sarcoma (KRAS), acinar cells dedifferentiate to an embryonic duct-like state and re-enter the cell cycle (red nuclei). Many mechanisms underlying this paligenotic process have been identified. Inflammation drives dedifferentiation, with transforming growth factor alpha (TGFα) and heparin-binding EGF-like growth factor (HB-EGF) ligands received by EGFR, which activates KRAS (Jhappan et al., 1990; Sandgren et al., 1990; Ardito et al., 2012). KRAS can activate protein kinase D1 (PRKD1) directly (Liou et al., 2015a), through Yes-associated protein 1 (YAP1) (Gao et al., 2013; Gruber et al., 2016) or through mitochondrial reactive oxygen species (mROS) (Liou et al., 2016). PRKD1 then activates the transcription factors pancreatic and duodenal homeobox 1 (PDX1), Notch1 (NICD) (Liou et al., 2015a), signal transducer and activator of transcription 3 (STAT3) (Miyatsuka et al., 2006) and nuclear factor kappa-B (NFκB), which drive dedifferentiation (Liou et al., 2016). KRAS also causes downregulation of MIST1 (Pin et al., 2001) and increases SRY-box 9 (SOX9) in acinar cells (Prevot et al., 2012; Grimont et al., 2015). KRAS can also signal through Rac family small GTPase 1 (RAC1) to activate phosphatidylinositol-4,5-bisphosphate 3-kinase (PI3K) (Heid et al., 2011; Baer et al., 2014; Wu et al., 2014), which activates the mitogen-activated protein kinase 1 (MEK)/extracellular regulated MAP kinase (ERK) signaling cascade (Collins et al., 2014). Myelocytomatosis oncogene (C-MYC) activity is increased in dedifferentiating acinar cells, inhibiting plastid transcription factor 1a (PTF1A) (Sanchez-Arevalo Lobo et al., 2017). Nuclear receptor subfamily 5 group A member 2 (NR5A2) also needs to be shut off for dedifferentiation to occur (von Figura et al., 2014). Once dedifferentiated, metaplastic cells can redifferentiate to acinar cells after the injury regresses, unless the presence of mutant, constitutively active KRAS or a similar mutation blocks redifferentiation (Collins et al., 2012), leading to metaplasia. Orange, transcription factors; yellow, other cellular proteins; red, extracellular signaling proteins; green, small molecules.

The pancreas is composed of two key secretory cell populations in distinct compartments. Hormone-secreting endocrine (Box 2) cells are housed in specialized islets of Langerhans (Elayat et al., 1995), whereas exocrine (Box 2) acinar cells are at the terminus of a network of ducts that carry their digestive enzymes to the duodenum (Fig. 3A). Zymogenic acinar cells in the adult pancreas parallel gastric chief cells, sharing function (digestive enzyme production), structure (a specialized secretory subcellular architecture) and gene expression [transcription factors that mediate the secretory cell architecture: X-box binding protein-1 (*Xbp1*) and *Mist1*] (Pin et al., 2001; Lo et al., 2017). Unlike the stomach, the adult pancreas lacks actively proliferating cells, necessitating mature cell plasticity whenever repair or proliferation are needed.

### Pancreatic plasticity

A rich literature has illustrated the inherent plasticity in both the adult endocrine and exocrine pancreatic lineages (Puri et al., 2015). Here, we focus on the acinar cells and acinar-to-ductal metaplasia (ADM), which fundamentally parallels gastric SPEM. As with SPEM, it is possible that sporadic or focal ADM arises spontaneously as a consequence of periodic physiological inflammation cycles that all tissues experience over the lifetime of an organism. ADM can also be induced experimentally in mice through cerulein (Box 2) injection, pancreatic duct ligation, pancreatectomy (Box 2) or genetic manipulation (Chan and Leung, 2007). During ADM, acinar cells switch to a proliferative state, adopting a more cuboidal morphology with a duct-like configuration (Blaine et al., 2010; Mills and Sansom, 2015). This process of mature acinar cells reverting to a regenerative duct-like state has been alternatively referred to as dedifferentiation, transdifferentiation or reprogramming in the published literature on ADM. For the sake of clarity and consistency, we will be referring to the changes undergone by these acinar cells as paligenosis, as the process appears highly conserved with the changes gastric chief cells and mature cells in other tissues undergo to become regenerative (Willet et al., 2018). Similarly to SPEM resembling the embryonic stomach, the cuboidal-ductal structures of ADM are also observed in multipotent progenitor cells in the embryonic pancreas (Jensen et al., 2005). Mouse models indicate that ADM is a process used by the pancreas to regenerate more acinar cells following large-scale injury (Jensen et al., 2005; Strobel et al., 2007; Fendrich et al., 2008) and, in certain cases, ADM cells may serve as SCs for other cell types (Zhou et al., 2008; Pan et al., 2013). Lineage tracing (De La O et al., 2008; Habbe et al., 2008; Morris et al., 2010a; Houbracken et al., 2011) and *in vitro* studies (Pinho et al., 2011) demonstrate that ADM cells largely arise via paligenosis of mature acinar cells. Thus, acinar cells are clearly capable of plasticity; however, it is possible that not all acinar cells have this property in equal measure, and there may be subpopulations of acinar cells with various levels of plasticity (Wollny et al., 2016).

### Pancreatic tumorigenesis

Similar to SPEM, ADM also correlates with increased risk for tumorigenesis. Indeed, in mouse cancer models and in human tissue, ADM seems to be the precursor lesion for pancreatic intraepithelial neoplasia (PanIN) (Hruban et al., 2005; Zhu et al., 2007), a clear precursor to pancreatic ductal adenocarcinoma (PDAC) (Ferreira et al., 2017). In humans, chronic pancreatitis (Box 2) resulting from alcohol consumption, smoking, familial conditions or spontaneous occurrence (Lowenfels et al., 1993; Malka et al., 2002; Hyun and Lee, 2014) increases risk for ADM, PanIN and PDAC (Guerra et al., 2007). Overactive mutant *KRAS* is present in the vast majority of PDACs (Almoguera et al., 1988) and at the PanIN stage (Klimstra and Longnecker, 1994). Genetically engineered mouse models faithfully recapitulate human PDAC tumorigenesis, with human-like ADM, PanIN and PDAC progression observed when constitutively active mutant *Kras*^G12D^ is expressed in the pancreas (Hingorani et al., 2005). Interestingly, although constitutively active mutant *Kras*^G12D^ is sufficient to cause ADM when expressed during development, both throughout the pancreas (Aguirre et al., 2003; Hingorani et al., 2003) or specifically in acinar cells (Grippo et al., 2003; Tuveson et al., 2006), a subsequent study by Guerra and colleagues demonstrated that induced expression of mutant *Kras*^G12V^ in mature acinar cells is not sufficient to induce paligenosis in acinar cells of adult mice. Rather, additional damage, such as cerulein injection, is required for mature acinar cells to progress to ADM and unmask the tumorigenic potential of the constitutively active KRAS (Guerra et al., 2007). Although *Kras*^G12D^ expressed in adult acinar cells can eventually be sufficient to drive acinar cell dedifferentiation to ADM and PanIN (Habbe et al., 2008), that is probably because sporadic injury and reprogramming events occur over the lifetime of an animal, inducing ADM, unmasking the mutant KRAS and causing clonal expansion. Accordingly, the process is still greatly accelerated by cerulein injections (Carrière et al., 2009), additional oncogenic mutations (Morris et al., 2010b) or even by destabilizing the mature acinar cell gene regulatory network via pancreas transcription factor 1 subunit alpha (*Ptf1a*) haploinsufficiency (Box 2) (Krah et al., 2015).

Although many studies demonstrate how KRAS activation can progress unidirectionally from ADM to PDAC, Collins et al. further explored this plasticity by conditionally expressing *Kras*^G12D^ until PanIN was induced and then stopping *Kras*^G12D^ expression. Most PanINs regressed within 2 weeks of mutant KRAS withdrawal, and green fluorescent protein tracing showed that the resulting healthy cells derived from the original acinar cells in which mutant KRAS expression was induced (Collins et al., 2012). Together, the results suggest that ADM, and even PanIN, are largely reversible, unless activated KRAS blocks redifferentiation, although aberrant KRAS activation itself is not sufficient to trigger the initial change, consistent with studies identifying *KRAS* mutations in healthy human pancreas (Lüttges et al., 1999). The data are consistent with our ‘cyclical hit’ model, in which the long-lived acinar cells might silently accrue mutations through cycles of dedifferentiation and redifferentiation until one final mutation, such as in *KRAS*, eventually inhibits their ability to redifferentiate from the ADM stage, locking them in a proliferative state that could lead to subsequent clonal expansion and progression to additional mutations and neoplasia (Fig. 1) (Mills and Sansom, 2015; Saenz and Mills, 2018).

Studying reprogramming (paligenosis) with respect to tumorigenesis in the pancreas carries some advantages over the stomach. The absence of SCs in the adult pancreas facilitates this, because increased proliferation during injury-induced regeneration must come from mature cells, whereas, in stomach and intestines, professional proliferating SC populations may also play a role in repair. Thus, studies of acinar cells are rapidly identifying the signaling pathways and genetic mechanisms governing the acinar cell changes, many of which will likely be shared in mechanisms of paligenosis across different organs (Means and Logsdon, 2016; Storz, 2017) (Fig. 3B). For example, we know that inflammation is key to ADM, with cyclooxigenase-2 (COX2)-mediated production of prostaglandin E being a key feature (Guerra et al., 2007; Liou et al., 2013). Inhibitors of macrophage activation and inflammation block ADM (Guerra et al., 2011; Liou et al., 2013). En route to ADM, acinar cells express intercellular adhesion molecule 1 (ICAM-1) to recruit additional macrophages (Liou et al., 2015b) and engage in aberrant epidermal growth factor receptor (EGFR) signaling (Jhappan et al., 1990; Sandgren et al., 1990; Ardito et al., 2012). Active KRAS also promotes ADM by suppressing Hippo kinases that would otherwise hold the mitogenic Yes-associated protein 1 (YAP1) (Gao et al., 2013; Gruber et al., 2016) transcription factor at bay. KRAS also promotes mitochondrial stress, creating mitochondrial reactive oxygen species that upregulate EGFR via polycystic kidney disease 1 (PKD1)/nuclear factor kappa-light-chain-enhancer of activated B cells (NFκB) (Liou et al., 2016), promoting ADM in a feed-forward loop.

Similar to gastric chief cells, mature acinar cells express MIST1 to maintain their subcellular secretory architecture (Pin et al., 2001; Lo et al., 2017). *Mist1*^─/─^ mice or mice with defective MIST1 exhibit both abnormal acinar cell maturation and ADM response (Pin et al., 2001; Zhu et al., 2004). Forced constitutive *Mist1* expression blocks ADM in spite of the presence of constitutively active *Kras*^G12D^ (Shi et al., 2013). Other transcription factors are keenly involved in acinar cell plasticity (Fig. 3B); notably, SOX9 (Furuyama et al., 2011; Prevot et al., 2012; Grimont et al., 2015), myelocytomatosis oncogene (C-MYC) (Sanchez-Arevalo Lobo et al., 2017) and Kruppel like factor 4 (KLF4) (Wei et al., 2016) promote ADM, whereas PTF1A (Krah et al., 2015; Benitz et al., 2016; Hoang et al., 2016; Jiang et al., 2016; Sanchez-Arevalo Lobo et al., 2017), nuclear receptor subfamily 5 group A member 2 (NR5A2) (Flandez et al., 2014; von Figura et al., 2014) and BHLH protein E47 (Kim et al., 2015) drive the cells to maintain a more acinar-like differentiated phenotype.

As mentioned above, the process whereby acinar cells convert to ADM parallels the process of gastric chief cells becoming SPEM. Thus, the paligenosis program for conversion of mature cells to regenerative cells is generally similar with the same stepwise sequence of autodegradation, SOX9/metaplastic gene expression and cell cycle re-entry. Accordingly, inhibition of autophagy/lysosomes or mTORC1 activity blocks the progression to ADM as it blocks progression to SPEM in the stomach (Willet et al., 2018). We expect that we are only at the beginning of understanding such shared processes in the progression of mature cells to metaplastic cell cycle re-entry in the pancreas, stomach and multiple other organs. Of course, some aspects of the process will likely be specific to acinar cells, such as the importance of the transcription factor PTF1A, because it supports pancreas-specific genes that are key to acinar cell function (Jiang et al., 2016). However, our current studies and the literature indicate a large swath of commonality, including the induction of SOX9 as a key feature of paligenosis. Indeed, SOX9 modulation as cells re-enter the cell cycle seems ubiquitous throughout the gastrointestinal (GI) tract (Van Landeghem et al., 2012; Roche et al., 2015). RAS signaling in dedifferentiation may be even more universal, as it plays a role not only in GI organs, but also in regenerating Schwann cells (Box 2) (Harrisingh et al., 2004), astrocytes and neurons (Friedmann-Morvinski et al., 2012).

## Conclusion

In the search for the cell of origin for epithelial cancers, investigators have long favored stem and progenitor cells as the likely culprits, owing to their constitutive proliferative capacity and supposed longevity (Fearon and Vogelstein, 1990; Vogelstein and Kinzler, 1993). However, before the rise of the scientific field of developmental biology, pathologists had considered three possible cancer cells of origin with relatively equal potential: (1) stem cells (or ‘mother cells’, as they were known over a century ago) (Adami, 1900); (2) ‘rests’, or cryptic embryonic cells which never fully differentiated in the adult; and (3) differentiated cells that can become proliferative again after potentially accumulating deleterious phenotypes. We are in the process of shifting our understanding of how tissues renew towards accepting that the more fluid/plastic notions of a century ago might describe reality more comprehensively than the rigid stem-cell-based unidirectional differentiation theories that predominated in the latter half of the twentieth century. A more nuanced understanding of stem and differentiated cells and their roles in tissue repair, now with molecular underpinnings of the underlying cellular processes, may help refine models of tumorigenesis. For example, intestinal SCs live for a shorter time than had been expected (Lopez-Garcia et al., 2010; Snippert et al., 2010). Thus, the longest-lived cells in many adult solid organs may actually be the differentiated populations. Thus, while many types of tumors may still ultimately arise from SCs (Visvader, 2011), the studies presented in this Review give cause to re-imagine the multi-hit model to include the potential contribution of fully differentiated post-mitotic cells such as gastric chief cells (Choi et al., 2016; Leushacke et al., 2017) and pancreatic acinar cells (Zhu et al., 2007) either as the direct cells of origin for tumors or as the sources for the stem/progenitor cells that go on to spawn cancer.

Opportunities to inhibit tumor initiation at the cell of origin may arise in multiple tissues if common pathways can be identified and manipulated to block their dedifferentiation. As a start, we can look at the many similarities between the stomach and pancreas discussed in this Review. Both systems begin with large, long-lived secretory cells that undergo paligenosis to give rise to smaller, simpler cells reminiscent of embryonic cell types. Both systems also lose similar maturity markers and share many signal-transducing and metaplastic genes, and both involve a role for inflammation (Table 2). Recent evidence indicates that paligenosis may be the process used during dedifferentiation of mature non-secretory cells in other organs as well, including liver and kidney (Willet et al., 2018), and evidence for proliferative dedifferentiation is also being delineated in diverse cell types, such as glia, warranting investigation into further mechanistic conservation (Friedmann-Morvinski and Verma, 2014). In part II of this Review (Burclaff and Mills, 2018), we will expand our scope and describe plasticity in the skin and intestine to continue to discuss its implications for tumorigenesis and to further highlight the conservation of plasticity-related genes and processes across tissues.

**Table 2.**
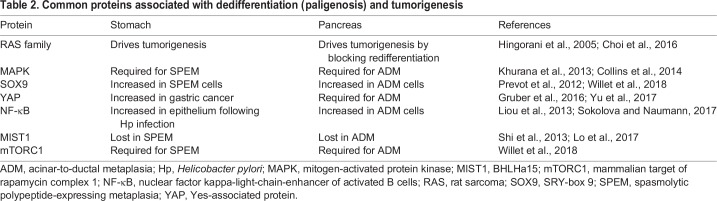
**Common proteins associated with dedifferentiation (paligenosis) and tumorigenesis**

Data from the stomach and pancreas support a model wherein mutations are acquired and stored through cycles of differentiation and dedifferentiation until a neoplastic mutation such as *KRAS* activation inhibits a paligenotic cell's ability to redifferentiate, which we describe as the cyclical hit model (Fig. 1B). This model might also help answer longstanding questions about tumor development. So-called ‘oncofetal’ gene expression in adult tumors has long puzzled oncologists (Uriel, 1979), with genes that are normally expressed only in early development becoming re-expressed in many tumors (Ährlund-Richter and Hendrix, 2014). Metaplastic gene re-expression is the second stage of paligenosis (Willet et al., 2018), consistent with the expression of embryonic genes being observed in ADM (Jensen et al., 2005), and the metaplastic stomach establishes morphology and cell types similar to the developing fetal gastric epithelium (Keeley and Samuelson, 2010; Osaki et al., 2010). It is thus likely that tumor cells (re)express these embryonic genes because the genes were reintroduced via a paligenosis event that occurred at some point in one of their cellular ancestors.

Clearly, we are only at the beginning of understanding how cell plasticity plays a role in tumorigenesis and how tumors can adapt to chemotherapy and radiation therapy. We will explore more angles in part II of this Review (Burclaff and Mills, 2018), but experimental data indicates that there may be an explosion of new ideas and potential therapeutic approaches as scientists begin to explore the concepts of cell plasticity and dedifferentiation, and the underlying conserved mechanisms and cellular processes, in more depth.
